# Structural Elucidation and In Silico-Aided Toxicity Prediction of Forced Degradation Products of Ginsenoside Re Using Ultra-High-Performance Liquid Chromatography Equipped with a Diode Array Detector and Charged Aerosol Detector (UHPLC-DAD-CAD) and Liquid Chromatography Coupled to a High-Resolution Mass Detector (LC-HRMS)

**DOI:** 10.3390/ijms252413231

**Published:** 2024-12-10

**Authors:** Yaqing Guo, Kai Wu, Haoran Yang, Xiaoyu Lin, Huiying Yang, Xianfu Wu

**Affiliations:** 1 National Institutes for Food and Drug Control, Beijing 102629, China; 2 College of Life Sciences and Health, Wuhan University of Science and Technology, Wuhan 430081, China

**Keywords:** ginsenoside Re, forced degradation products, charged aerosol detector, LC-HRMS, mass fragmentation pathway, toxicity

## Abstract

Ginsenoside Re was the major bioactive component found rich in *Panax ginseng* C. A. Meyer, which exerted excellent cardiovascular protection, anti-inflammatory, and anti-oxidation effects. The generation of unexpected degradation products (DPs) may influence the therapeutic effect of Re, or even bring toxic effects to patients. However, to date, only a few reports were available about the stability of Re. The present study aims to systematically investigate the degradation behaviors of Re under different stress conditions, including hydrolysis (acidic, basic, and neutral), oxidation, humidity, thermal, and photolytic (ultraviolet and visible light) conditions. A total of thirteen DPs were putatively identified, and among them, nine were discovered for the first time in our study. The results showed that Re was sensitive to exposure to acidic, basic, and oxidation conditions. It underwent a series of chemical degradation reactions, including deglycosylation, dehydration, addition, oxidation at the double bond, and isomerization under various stress conditions. Structural characterization of these DPs was carried out by UHPLC-DAD-CAD and LC-LTQ/Orbitrap. A plausible mechanism of their formation was proposed to support the structures of all DPs of Re. In silico toxicity prediction and metabolism behavior assessment were done by Derek Nexus and Meteor Nexus software. Re and DP-1 to DP-6 were predicted to possess potential skin irritation/corrosion toxicity. DP-11 and DP-12 bear the potential for carcinogenicity, mutagenicity, irritation, hepatotoxicity, and skin sensitization. The observation of these DPs updates our knowledge regarding the stability of Re, which provides valuable information for quality control and to choose suitable storage conditions.

## 1. Introduction

**Figure 1:**
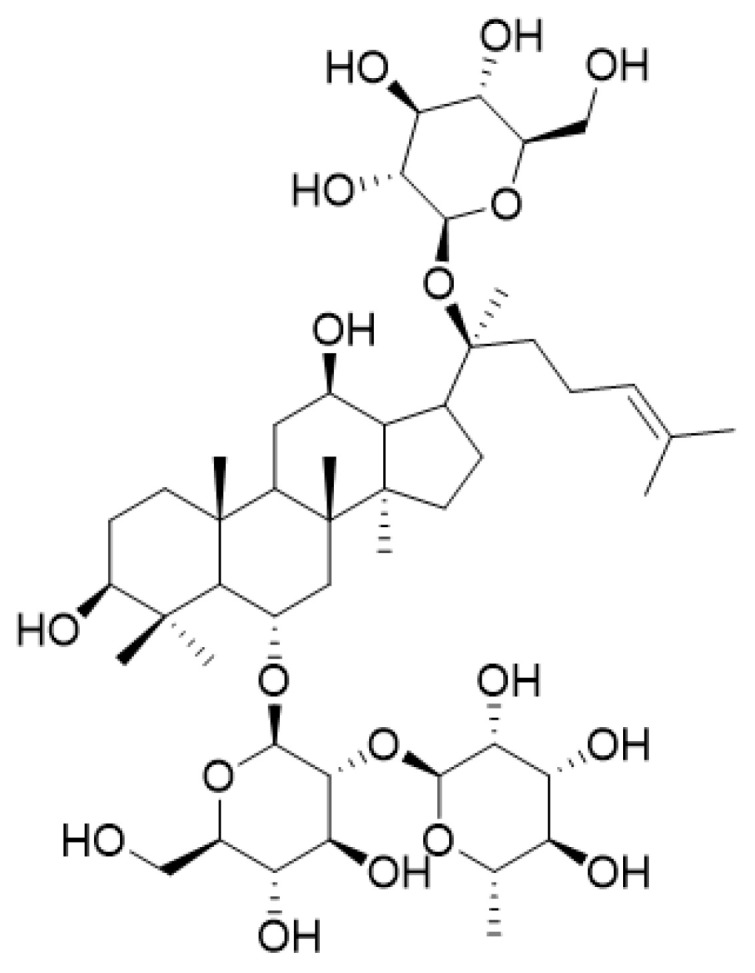
Chemical structure of ginsenoside Re.

Ginsenosides, belonging to triterpenoid glycoside compounds, have widely been considered the major bioactive constituents of *Panax ginseng* C. A. Meyer (*P. ginseng* or ginseng). They are usually divided into three types based on their aglycones and positions of glycosidic linkages, namely the 20(*S*)-protopanaxadiol type (PPD), 20(*S*)-protopanaxatriol type (PPT), and oleanane type (OA) [1,2,3,4,5]. Ginsenoside Re (shorted as Re), known as a PPT-type saponin, is a unique tetracyclic triterpenoid derivative with one rhamnosyl and two glucosyl groups at the C-6 and/or C-20 positions (Figure 1). It has been proven to possess a variety of beneficial effects, including anti-oxidation, anti-inflammatory, anti-tumor, anti-platelet aggregation, anti-arrhythmia, cardiovascular protection, blood glucose reduction, and so on [6,7,8,9].

Given its remarkable biological activities, Re has long been regarded as the quality control marker for *P. ginseng*, and its relevant preparations have been collected in the Chinese Pharmacopoeia (version 2020), European Pharmacopoeia, British Pharmacopoeia, and USA Herbal Medicines Compendium [10,11]. The stability and purity of Re is crucial for the quality control and evaluation of ginseng and its related products in the herbal market, food, and cosmetic industries. Moreover, the production of unwanted degradation products (DPs) may influence the therapeutic effect of Re, or even bring toxic effects to patients. However, knowledge of the stability of Re remains limited, and only a few reports were available [12]. Thus, systematic stress studies were urgent to conduct to ensure the quality, therapeutic efficacy, and safety of Re during transportation, storage, and usage.

Herein, a comprehensive forced degradation study of Re was performed under hydrolysis (acidic, basic, and neutral), oxidation (30% H_2_O_2_), humidity, thermal, and photolytic (ultraviolet and visible light) stress conditions using ultra-high-performance liquid chromatography equipped with a diode array detector and charged aerosol detector (UHPLC-DAD-CAD) and liquid chromatography coupled to a high-resolution mass detector (LC-HRMS). A total of thirteen DPs were detected, and nine of them were newly discovered in our study. The plausible structures of all DPs and degradation mechanisms of Re were proposed. Furthermore, the potential toxicological impact and in vivo metabolism fates of Re and its DPs were predicted by Derek Nexus and Meteor Nexus software, respectively. The observation of these DPs updates our knowledge regarding the stability of Re, which provides valuable information for quality control and to choose suitable storage conditions.

## 2. Results

### 2.1. Forced Degradation Behaviors of Re

**Figure 2:**
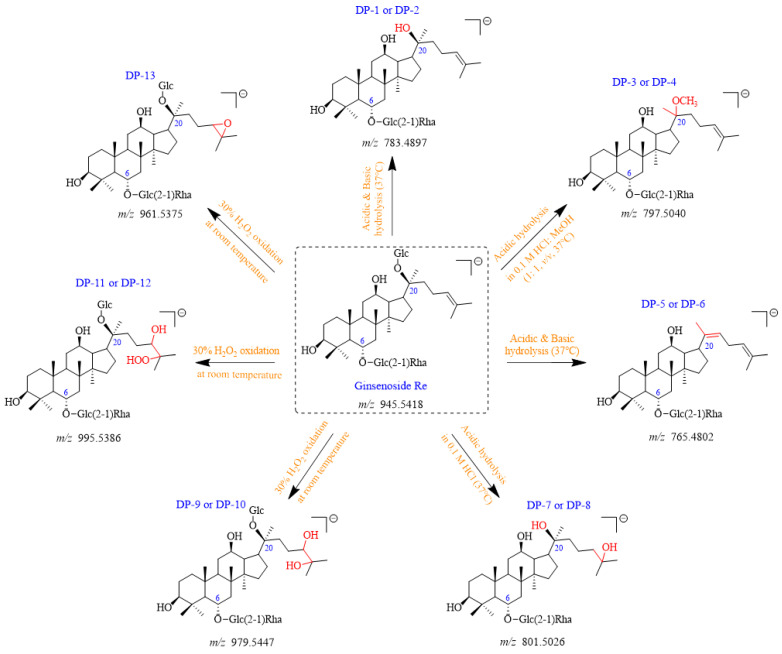
Plausible chemical structures of all DPs from ginsenoside Re. Glc, glucosyl; Rha, rhamnosyl.

**Figure 3:**
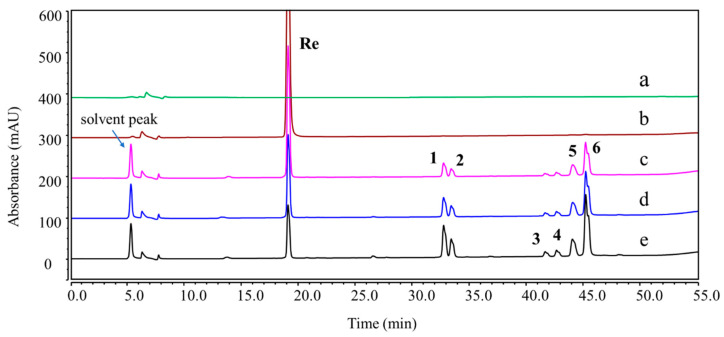
Overlay chromatograms of Re and its degradation products (DPs) under acidic hydrolysis using UHPLC-UV (203 nm). (a) Blank; (b) control sample (pure ginsenoside Re); (c–e) acidic hydrolysis (0.1 M HCl:MeOH = 1:1, 37 °C) for 1, 3, and 6 h.

**Figure 4:**
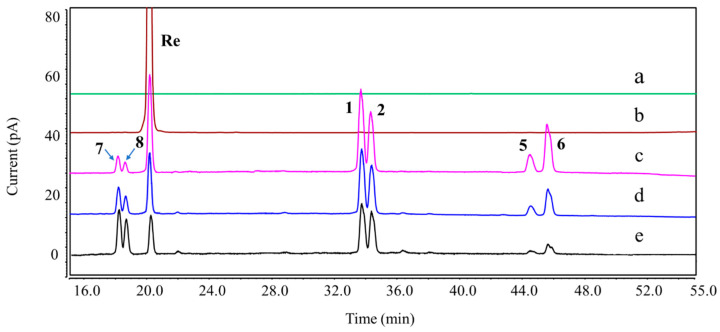
Overlay chromatograms of Re and its DPs under acidic hydrolysis using UHPLC-CAD. (a) Blank; (b) Re; (c–e) acidic hydrolysis (0.1 M HCl, 37 °C) for 1, 3, and 6 h.

**Figure 5:**
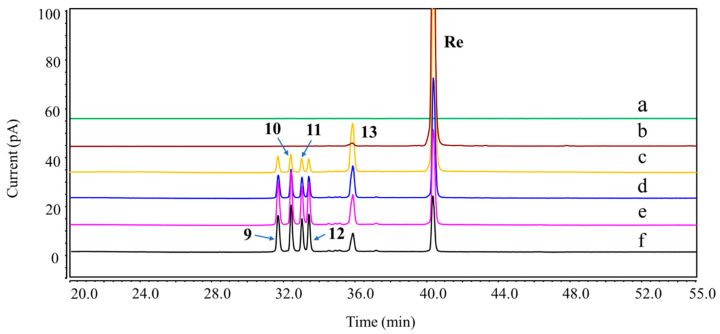
Overlay chromatograms of Re and its DPs under oxidation stress using UHPLC-CAD. (a) Blank; (b) Re; (c–f) 30% H_2_O_2_ for 1, 3, 6, and 8 h at room temperature.

A total of thirteen DPs were generated from Re, among which nine were first detected. Their plausible structures are displayed in Figure 2. These DPs were mainly formed under acidic, basic, and H_2_O_2_ oxidation stress conditions. DP-1, DP-2, DP-5, and DP-6 were found in both acidic and basic hydrolysis (Figure S1). Four other DPs were formed under different acidic conditions. DP-3 and DP-4 were only observed in 0.1 M HCl: MeOH (1:1, *v*/*v*) at 37 °C (Figure 3), whereas DP-7 and DP-8 were only formed in 0.1 M HCl at 37 °C (Figure 4). Under the oxidation stress condition, we found the generation of DP-9 to DP-13 (Figure 5). Among them, DP-7 to DP-13 can only be detected by the CAD detector owing to the absence of chromophore groups. No major DPs of Re were formed when exposed to neutral hydrolysis, high humidity, high temperature, and photolytic stress conditions. The overlayed chromatograms of Re under these conditions are shown in Figures S2–S4.

### 2.2. Mass Fragmentation Behavior of Re and Structural Characterization of Its DPs

Compared to the positive ion mode, Re exhibited a higher response in the negative ion mode. Hence, the mass spectra of Re and its DPs were acquired by HPLC-ESI-LTQ/Orbitrap in negative ionization mode. We first analyzed the mass fragmentation pathway of Re. The chemical structures of all DPs were then proposed by comparing their molecular masses and mass spectrometric fragmentation patterns with that of Re. The most plausible structures for all DPs are presented in Figure 2. All observed accurate masses, mass errors in ppm, the best possible molecular formulas, ring plus double bonds (RDBs), and major MS/MS fragment ions are listed in Table 1. The structural characterization for all DPs is discussed below.

#### 2.2.1. Re (*m*/*z* 945.5418)

In this study, Re gave an [M-H]^−^ ion peak at *m*/*z* 945.5418 (C_48_H_81_O_18_^−^) and formic acid adduct [M + HCOO]^−^ at *m*/*z* 991.5447 (C_49_H_83_O_20_^−^), which dissociated into product ions at *m*/*z* 799.4806 and *m*/*z* 783.4860 due to the loss of C-6 rhamnose residue (Rha) and C-20 glucose residue (Glc). The product ion at *m*/*z* 783.4860 further yielded *m*/*z* 765.4754 with a loss of H_2_O. The fragment ion at *m*/*z* 637.4287 was formed by the elimination of Glc at C-20 or C-6 from *m*/*z* 799.4806. The *m*/*z* 637.4287 further generated two fragment ions of *m*/*z* 619.4181 and *m*/*z* 475.3766 with the loss of H_2_O as well as Glc at C-6 or C-20, respectively. The MS/MS fragmentation pattern of Re is shown in Figures S5 and S6, which was consistent with previous studies [1,13,14,15].

#### 2.2.2. DP-1 and DP-2 (*m*/*z* 783.4897)

**Figure 6:**
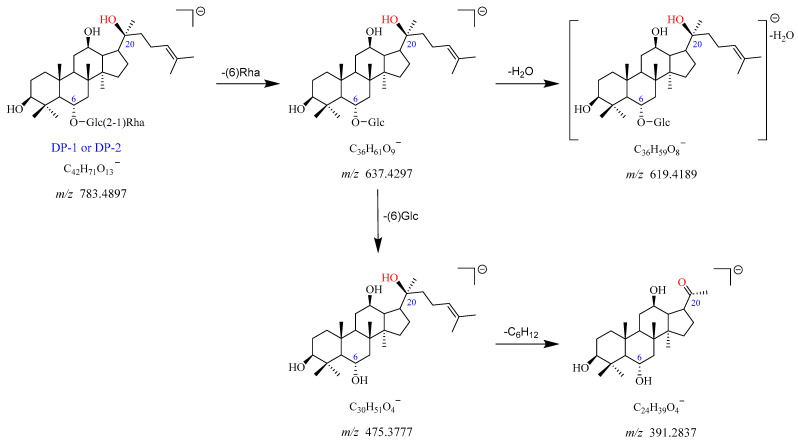
Mass fragmentation pathway of DP-1 and DP-2.

The precursor ion of DP-1 was observed at *m*/*z* 783.4897 [M-H]^−^, which corresponds to the chemical formula of C_42_H_71_O_13_^−^. The *m*/*z* 829.4921 was assigned as the [M + HCOO]^−^ adduct ion of DP-1 with the chemical formula of C_43_H_73_O_15_^−^. The fragment ion at *m*/*z* 637.4297 arose from *m*/*z* 783.4897 after the loss of Rha (C6). The *m*/*z* 637.4297 further fragmented into *m*/*z* 619.4189 and *m*/*z* 475.3777 with the elimination of H_2_O and Glc (C6), respectively. The product ion at *m*/*z* 391.2837 yielded from *m*/*z* 475.3777 due to the loss of its side chain (-C_6_H_12_). The proposed fragmentation pathway for DP-1 is displayed in Figure 6 and Figure S7. Based on these data, DP-1 was identified as the deglycosylated product of Re after the removal of Glc (C20), namely ginsenoside Rg_2_. It was further confirmed by the reference standard. As DP-1 and DP-2 showed the same mass and similar mass fragmentation patterns, they may be the structural isomers of each other, which was in line with previous studies [12,16].

#### 2.2.3. DP-3 and DP-4 (*m*/*z* 797.5040)

**Figure 7:**
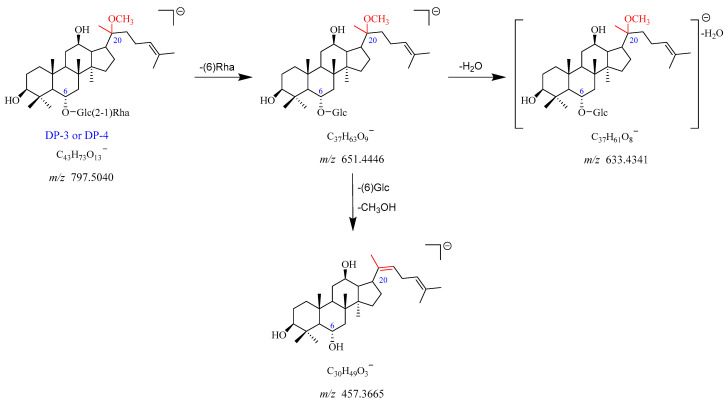
Mass fragmentation pathway of DP-3 and DP-4.

DP-3 showed the [M-H]^−^ and [M + HCOO]^−^ adduct ion peaks at *m*/*z* 797.5040 and *m*/*z* 843.5076, which matched the formulas of C_43_H_73_O_13_^−^ and C_44_H_75_O_15_^−^, respectively. The *m*/*z* 797.5040 fragmented into *m*/*z* 651.4446 by elimination of Rha (C6). The product ion at *m*/*z* 651.4446 further yielded a fragment ion of *m*/*z* 633.4341 after the neutral loss of H_2_O. In addition, the *m*/*z* 651.4446 can also dissociate into *m*/*z* 457.3665 upon the removal of Glc (C6) and one molecule of CH_3_OH. The fragmentation pathway of DP-3 is depicted in Figure 7 and Figure S8. Thus, DP-3 was proposed to be the product of Re with deglycosylation, dehydration, and addition with MeOH. Likewise, DP-3 and DP-4 shared identical mass and similar MS/MS fragmentation patterns, indicating that they were a pair of isomers.

#### 2.2.4. DP-5 and DP-6 (*m*/*z* 765.4802)

**Figure 8:**
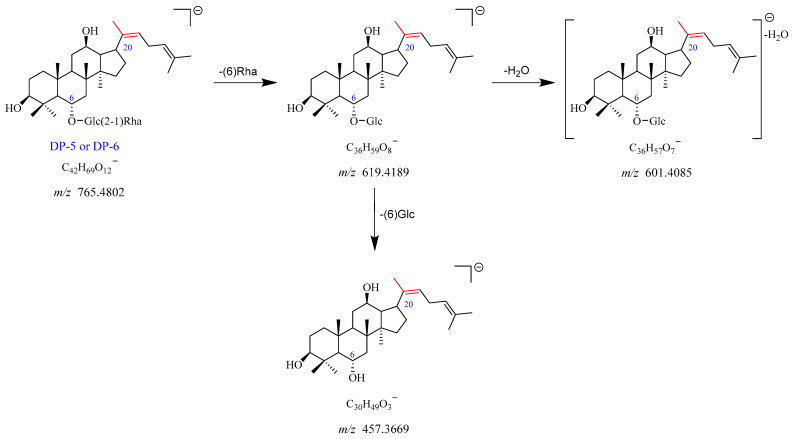
Mass fragmentation pathway of DP-5 and DP-6.

The precursor ion peak [M-H]^−^ of DP-5 was observed at *m*/*z* 765.4802, with an [M + HCOO]^−^ adduct ion peak at *m*/*z* 811.4828. The corresponding chemical formulas of [M-H]^−^ and [M + HCOO]^−^ were C_42_H_69_O_12_^−^ and C_43_H_71_O_14_^−^, respectively. The daughter ion at *m*/*z* 619.4189 originated from *m*/*z* 765.4802 through the elimination of Rha (C6). The *m*/*z* 619.4189 further dissociated into two product ions at *m*/*z* 601.4085 and *m*/*z* 457.3669 by the loss of H_2_O and Glc (C6), respectively. The proposed fragmentation pathway for DP-5 is shown in Figure 8 and Figure S9. Based on the above evidence, DP-5 was supposed to be the deglycosylation and dehydration product of Re. DP-6 was an isomer of DP-5, which was in accordance with previously studies [12].

#### 2.2.5. DP-7 and DP-8 (*m*/*z* 801.5026)

**Figure 9:**
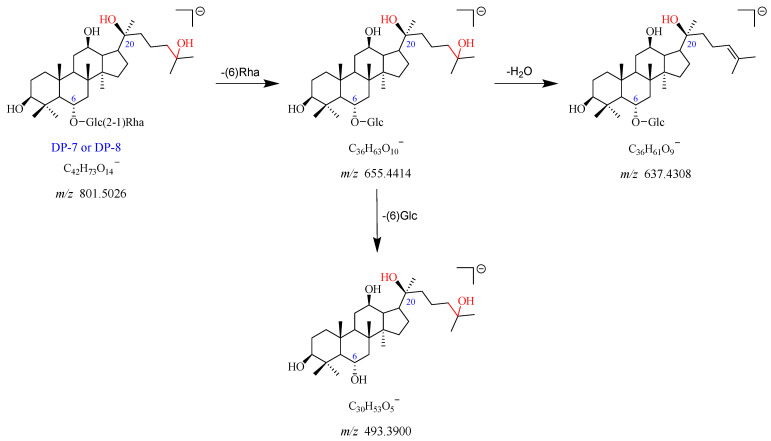
Mass fragmentation pathway of DP-7 and DP-8.

DP-7 exhibited the [M-H]^−^ and [M + HCOO]^−^ adduct ion peaks at *m*/*z* 801.5026 and *m*/*z* 847.5050, which matched the formulas of C_42_H_73_O_14_^−^ and C_43_H_75_O_16_^−^, respectively. The formation of the fragment ions at *m*/*z* 655.4414 could be attributed to the loss of Rha (C6) from the *m*/*z* 801.5026. The *m*/*z* 655.4414 further yielded two fragment ions at *m*/*z* 637.4308 with the neutral loss of one molecule of H_2_O, and at *m*/*z* 493.3900 by the elimination of Glc (C6). The proposed fragmentation pathway for DP-7 is given in Figure 9 and Figure S10. Furthermore, DP-7 did not show a UV absorption peak. According to these data, DP-7 was tentatively proposed as the product of Re of deglycosylation and addition with H_2_O. As the chromatographic and mass spectrometric spectra of DP-8 were the same as that of DP-7, we suspected that DP-8 was an isomer of DP-7.

#### 2.2.6. DP-9 and DP-10 (*m*/*z* 979.5447)

**Figure 10:**
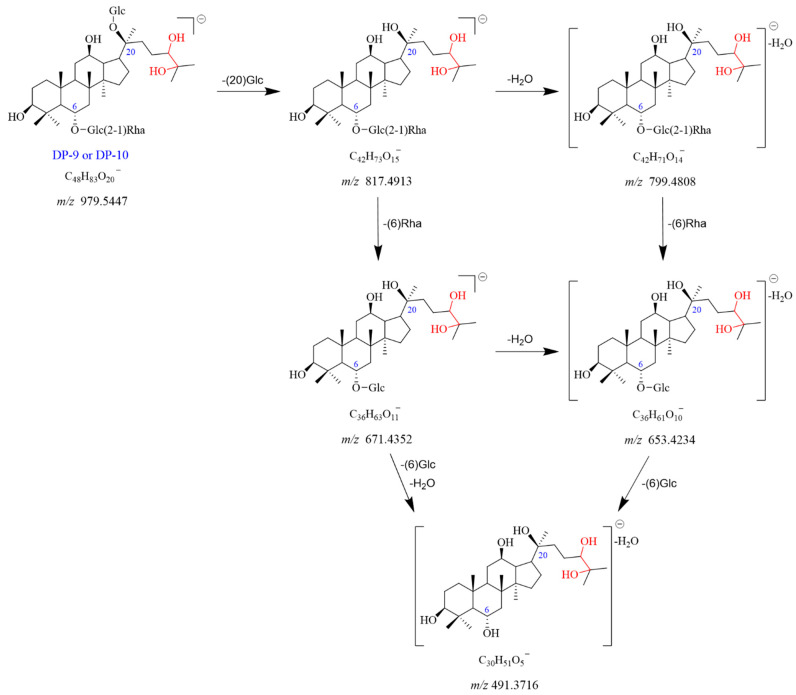
Mass fragmentation pathway of DP-9 and DP-10.

A molecular ion peak [M-H]^−^ of DP-9 was found at *m*/*z* 979.5447 with the chemical formula C_48_H_83_O_20_^−^. It showed the [M + HCOO]^−^ adduct ion peak at *m*/*z* 1025.5494, which matched the formula of C_49_H_85_O_22_^−^. The product ion observed at *m*/*z* 817.4913 was formed by the loss of Glc (C20) from the *m*/*z* 979.5447. The *m*/*z* 817.4913 dissociated into two fragment ions at *m*/*z* 799.4808 and *m*/*z* 671.4352, caused by the elimination of H_2_O and Rha (C6), respectively. The fragment ion at *m*/*z* 653.4234 could be formed from *m*/*z* 799.4808 by a loss of Rha (C6) or from *m*/*z* 671.4352 with a loss of H_2_O. The *m*/*z* 491.3716 originated from *m*/*z* 671.4352 with the elimination of Glc (C6) and H_2_O. It also can dissociate from *m*/*z* 653.4234 with a loss of Glc (C6). DP-9 had no UV absorption. Based on the above data, DP-9 was tentatively identified as the oxidation product of Re (Re + H_2_O_2_). DP-9 and DP-10 were isomers of each other. The proposed fragmentation pathway for DP-9 and DP-10 is illustrated in Figure 10 and Figure S11.

#### 2.2.7. DP-11 and DP-12 (*m*/*z* 995.5386)

**Figure 11:**
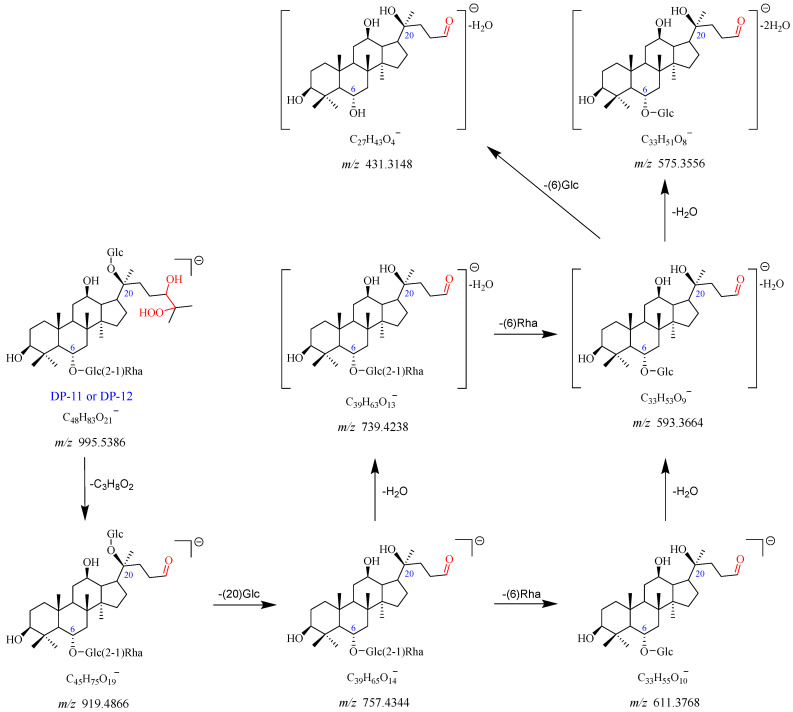
Mass fragmentation pathway of DP-11 and DP-12.

DP-11 displayed the ion peaks [M-H]^−^ and [M + HCOO]^−^ at *m*/*z* 995.5386 and *m*/*z* 1041.5441, which align with the chemical formulas C_48_H_83_O_21_^−^ and C_49_H_85_O_23_^−^, respectively. The product ion at *m*/*z* 919.4866 dissociated from *m*/*z* 995.5386 with a loss of C_3_H_8_O_2_. It went through a further loss of Glc (C20) to produce a fragment ion at *m*/*z* 757.4344. The *m*/*z* 757.4344 dissociated into two product ions at *m*/*z* 739.4238 and *m*/*z* 611.3768 with the elimination of H_2_O and Rha (C6), respectively. The *m*/*z* 593.3664 originated from *m*/*z* 739.4238 by a loss of Rha (C6) or *m*/*z* 611.3768 by a loss of one molecule of H_2_O. Furthermore, the *m*/*z* 593.3664 further dissociated into fragment ions at *m*/*z* 575.3556 and *m*/*z* 431.3148, caused by the elimination of one molecule of H_2_O and Glc (C6), respectively. DP-11 also had no UV absorption. Therefore, DP-11 was speculated to be the peroxidation product of Re (Re + H_2_O_3_). DP-11 and DP-12 were a pair of isomers. The fragmentation pathway of DP-11 and DP-12 is shown in Figure 11 and Figure S12.

#### 2.2.8. DP-13 (*m*/*z* 961.5375)

**Figure 12:**
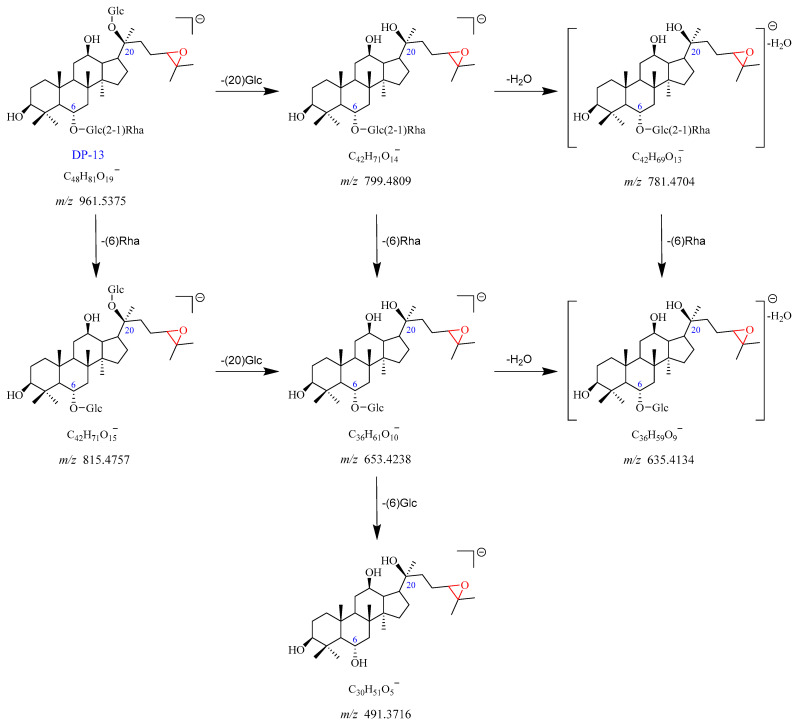
Plausible mass fragmentation pathway of DP-13.

The precursor ion peak [M-H]^−^ of DP-13 was observed at *m*/*z* 961.5375, with an [M + HCOO]^−^ adduct ion peak at *m*/*z* 1007.5393. The corresponding chemical formulas of [M-H]^−^ and [M + HCOO]^−^ were C_48_H_81_O_19_^−^ and C_49_H_83_O_21_^−^, respectively. It was 16 Da higher than Re, suggesting that DP-13 was the monooxygenation product of Re. The *m*/*z* 961.5375 yielded two fragment ions at *m*/*z* 815.4757 after the loss of Rha (C6), and at *m*/*z* 799.4809 through the elimination of Glc (C20). The product ion observed at *m*/*z* 781.4704 was formed by the loss of H_2_O from the *m*/*z* 799.4809. The *m*/*z* 653.4238 arose from *m*/*z* 815.4757 by a loss of Glc (C20), which further dissociated into fragment ion at *m*/*z* 635.4134 with the elimination of one molecule of H_2_O. In addition, the *m*/*z* 653.4238 and *m*/*z* 635.4134 also can be formed by a loss of Rha (C6) from *m*/*z* 799.4809 and *m*/*z* 781.4704, respectively. The product ion at *m*/*z* 491.3716 dissociated from *m*/*z* 653.4238 with a loss of Glc (C6). DP-13 has no UV absorbance. Based on these data, DP-13 was tentatively proposed as the epoxidation product of Re (Re + O). The proposed fragmentation pathway for DP-13 is indicated in Figure 12 and Figure S13.

### 2.3. In Silico Toxicity and Metabolic Behavior Assessment

The prediction of toxicity profiles for Re and its DPs are listed in Table S1. DP-1 to DP-6, as well as Re, showed potential skin irritation/corrosion in different species, which may be attributed to the structure of the terpenoid. DP-11 and DP-12 were predicted to have effects of carcinogenicity, mutagenicity, irritation, hepatotoxicity, and skin sensitization owing to the presence of the structure of peroxide. The structures of the other DPs did not contain any toxicophores to cause an alert. The predicted metabolic behaviors of Re and its DPs are summarized in Table S2. The main biotransformation pathways of phase I were allylic hydroxylation, epoxidation of 1,1,2-trisubstituted alkenes, oxidative *O*-demethylation, hydroxylation of methyl carbon adjacent to an aliphatic ring, hydroxylation of terminal methyl, and vicinal diols from epoxides. The major pathway of phase II was glucuronidation of primary and secondary aliphatic and benzylic alcohols. The metabolic enzymes involved in CYP450, EH, and UGT. Thus, the formation of DP-1 to DP-6, as well as DP-11 and DP-12, under acidic hydrolysis and oxidation conditions should be cautiously monitored.

## 3. Discussion

The results uncovered that Re showed extensive degradation under acidic, basic, and oxidation conditions but was stable under the other conditions. Specifically, acid and oxidate conditions facilitated the degradation process. These DPs generally appeared in the forms of several pairs of isomers, such as DP-1 and DP-2. A total of six pairs of isomer compounds were detected in this study. Under acidic and basic conditions, Re was prone to initially lose the C-20 sugar (Glc), and further lose another single molecule of H_2_O. The degradation rate in the basic condition was slower as compared to that in acidic hydrolysis. Additionally, Re was prone to undergo addition reactions at the C=C position in the side chain under the acidic condition. The types of DPs generated vary with the addition time of cosolvent MeOH, and Re underwent an addition reaction with MeOH or H_2_O when MeOH was added before or after heating, respectively. This would result in the production of DPs without UV absorption. The results indicated that alcoholic solvents used as cosolvents can lead to the formation of more DPs under the acidic hydrolysis condition. To minimize the formation of the unnecessary DPs, utilizing an aprotic solvent, such as tetrahydrofuran (THF), *N*,*N*-dimethylformamide (DMF), or dimethyl sulfoxide (DMSO), as a cosolvent may be a better choice.

**Figure 13:**
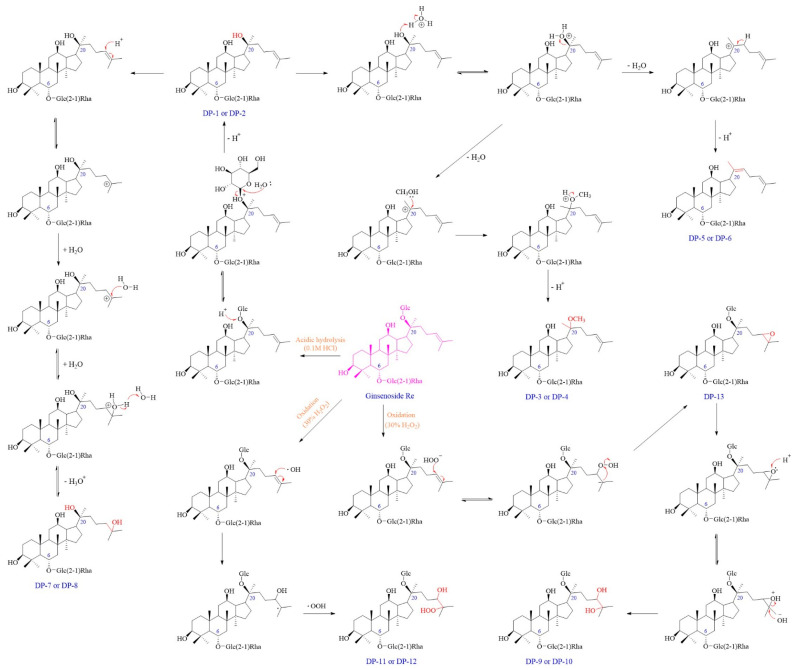
Plausible degradation mechanisms for the formation of DPs of Re under various forced degradation conditions.

Similarly, Re tended to experience an oxidation reaction under the oxidative stress condition. We observed that DP-9 to DP-13 with no UV absorption were generated under this condition, and their elution times in the chromatogram were earlier than that of Re. The results further confirmed the introduction of an oxygen atom and that the exact position was at the double bond on the C-17 side chain. Postulated degradation mechanisms of all DPs are outlined in Figure 13. Our studies suggested that Re was susceptible to acidic, alkaline, and oxidative stress conditions. Therefore, it is recommended to avoid exposure to acids, bases, and oxides during the storage and usage of Re.

Due to the simplicity of the degradation condition (under the pH 5.0 ± 0.1 environment), only DP-1, DP-2, DP-5, and DP-6 were reported in previous studies [12]. For better insight into the stability and degradation pathways of Re, it is necessary to conduct comprehensive stress studies under milder or even severe degradation conditions. Nine novel DPs were thus discovered for the first time in our study, which not only provides useful information for the quality control of Re during its transportation, storage, and usage, but also contributes to the design of Re-based potential drugs with both safety and efficacy. However, computer-aided toxicity prediction can only alert us to the potential safety risks of these DPs. The prediction accuracy largely depends on the size and diversity of datasets, the quality of available toxicity data, the choice of modeling methods, and so on [17]. Further research is needed to acquire reliable and sufficient toxicity experimental data in vitro and in vivo for the safety assessment of these DPs.

## 4. Materials and Methods

### 4.1. Chemicals and Reagents

Ginsenoside Re (96.9%, Batch No.110754-202330) was acquired from the National Institutes for Food and Drug Control (Beijing, China). LC-MS-grade methanol (MeOH) and acetonitrile (ACN) were purchased from Dikma Technologies Inc. (Foothill Ranch, CA, USA). Analytical-grade hydrochloric acid (HCl), sodium hydroxide (NaOH), and 30% hydrogen peroxide (H_2_O_2_) were obtained from Sinopharm Chemical Reagent Co., Ltd. (Shanghai, China). HPLC-grade formic acid (98%) was provided by Shanghai Aladdin Biochemical Technology Co., Ltd. (Shanghai, China). Ultrapure water was generated from a Milli-Q plus system (Millipore, MA, USA).

### 4.2. Sample Preparation

The sample solution of Re (1.0 mg/mL) for UHPLC analysis was prepared by dissolving 1.5 mg of Re in various solutions, used in forced degradation tests. All the stress samples were subjected to further dilution to give a final concentration of 500 ng/mL for LC-HRMS analysis. MeOH/water (50:50, *v*/*v*) was utilized as the diluent solution.

### 4.3. UHPLC-DAD-CAD Chromatographic Condition

The stress samples were analyzed using Thermo Scientific^TM^ Vanquish^TM^ UHPLC equipment equipped with a diode array detector (DAD) and charged aerosol detector (CAD). Chromatographic separation was carried out on a Waters Symmetry^®^ C18 column (4.6 × 250 mm, 5 μm, Waters, Milford, MA, USA) at 30 °C. The mobile phase consisted of A (0.1% formic acid/water) and B (ACN). The gradient elution was as follows: 0–25 min, 20–40% B; 25–45 min, 40–60% B; 45–50 min, 60–95% B; 50–57 min, 95% B; 57–58 min, 95–20% B; 58–80 min, 20% B. The more suitable gradient program for oxidation samples was as follows: 0–20 min, 10–13% B; 20–50 min, 13–60% B; 50–57 min, 60–95%; 57–63 min, 95% B; 63–70 min, 95–10% B; 70–80 min, 10% B. The chromatograms were collected from the DAD detector with a wavelength of 203 nm or the CAD detector. The autosampler temperature was controlled at 10 °C. The flow rate was 0.4 mL/min, and the injection volume was 10 μL for UHPLC analysis.

### 4.4. LC-HRMS Condition

Chromatographic analysis was conducted on the Surveyor Plus HPLC system with a PDA detector (Thermo Fisher Scientific, San Jose, CA, USA). The DPs were separated using an Agilent InfinityLab Poroshell 120 EC-C18 column (2.1 × 100 mm, 2.7 μm, Agilent, Santa Clara, CA, USA) maintained at 30 °C. The gradient elution was as follows: 0–10 min, 20–40% B; 10–25 min, 40–60% B; 25–35 min, 60–95% B; 35–35.10 min, 95–20% B; 35.10–42 min, 20% B. For oxidation samples, the gradient program was set as follows: 0–10 min, 10–13% B; 10–25 min, 13–60% B; 25–35 min, 60–95%; 35–35.10 min, 95–10% B; 35.10–42 min, 10% B. The flow rate was 0.3 mL/min. Other parameters were the same as those in the Section 4.3.

High-resolution mass spectra for structural characterization were acquired by the LTQ Orbitrap XL^TM^ system (Thermo Fisher Scientific, San Jose, CA, USA) with an electro-spray ionization (ESI) source. Samples were detected in the negative mode. The MS settings were optimized as follows: sheath gas, 35 arb; auxiliary gas, 10 arb; sweep gas, 1 arb; spray voltage, 5 kV; capillary voltage, −35 V; tube lens, −200 V; capillary temperature and auxiliary gas heater temperature, 350 °C; *m*/*z*, 200–1500 Da; collision energy, 35 V; analyzer, FT-HRMS; resolution, 60,000 for MS, 30,000 for MS/MS. High-purity nitrogen (N_2_) and helium (He) were used as the nebulizing gas and collision gas, respectively.

### 4.5. Forced Degradation Studies

Forced degradation studies of Re were performed according to the ICH Q2 (R1) guidelines. The stability of Re was tested under various stress conditions: hydrolysis (acidic, basic, and neutral), oxidation with 30% H_2_O_2_, high temperature, high humidity, and photolysis in UV light and visible light. There were two samples prepared for acidic or basic stress studies. Sample 1: 1.5 mg of Re was treated with 0.5 mL 0.1 M HCl/1.0 M NaOH and 0.5 mL MeOH, then placed in a drying oven at 37 °C for 1, 3, and 6 h. After that, 0.5 mL 0.1 M NaOH/1.0 M HCl was added to neutralize the solution. Sample 2: 1.5 mg of Re was only treated with 0.5 mL 0.1 M HCl/1.0 M NaOH and placed in an oven at 37 °C. After 1, 3, and 6 h, the solution was mixed with 0.5 mL MeOH and 0.5 mL 0.1 M NaOH/1.0 M HCl. For neutral hydrolysis and oxidative stress studies, 1.5 mg of Re was treated with 1.5 mL MeOH and 30% H_2_O_2_, respectively. The other stress studies of Re were carried out in the solid state. The detailed degradation conditions and behaviors of Re are listed in Table 2.

### 4.6. Toxicity Risk Assessment and Metabolic Behavior Prediction of Re and Its DPs

The safety and efficacy of Re will be put at risk once the in vivo metabolic behavior of its DPs changes. Derek Nexus (version 6.3.0) and Meteor Nexus software (version 3.2.0, Lhasa Limited, Leeds, UK) were utilized as in silico tools to predict the potential toxicity and metabolic fate of Re and its DPs. The computational logic and principles for prediction behind the software were reported in our previous study [18].

## 5. Conclusions

The present study first systematically investigated the degradation behaviors of ginsenoside Re under hydrolysis (acidic, basic, and neutral), oxidation, humidity, thermal, and photolytic (ultraviolet and visible light) conditions. Re showed significant degradation when exposed to acidic, alkaline, and oxidative stress conditions. A total of thirteen DPs were putatively identified, and nine of them were discovered for the first time in our study. UHPLC-DAD-CAD and LC-HRMS were utilized to characterize the probable structures of these DPs. Additionally, postulated degradation pathways and mechanisms of all DPs were also outlined in this study. The results revealed that Re mainly experienced several chemical degradation reactions, including deglycosylation, dehydration, addition, oxidation at the double bond, and isomerization under various stress studies. The predicted toxicity profiles indicated that Re, as well as DP-1 to DP-6, showed potential skin irritation/corrosion effects. DP-11 and DP-12 bear the potential for carcinogenicity, mutagenicity, irritation, hepatotoxicity, and skin sensitization. In conclusion, our findings provide valuable insights into the chemical stability of ginsenoside Re, and may contribute to ensuring the quality, therapeutic efficacy, and safety of Re during transportation, storage, and usage.

## Figures and Tables

**Table 1:** LC-MS/MS data for fragmentation profile of DPs (1–13) along with their possible molecular formulas.

Degradation Products (DPs)	Observed Mass (*m*/*z*)	Theoretical Mass (*m*/*z*)	Error (ppm)	Best Probable Molecular Formulas	RDBs ^a^	Major Fragments (Chemical Formulas)
DP-1 and DP-2	783.4897 [M-H]^−^	783.4889	0.997	C_42_H_71_O_13_^−^	7.5	637.4297 (C_36_H_61_O_9_^−^), 619.4189 (C_36_H_59_O_8_^−^), 475.3777 (C_30_H_51_O_4_^−^), 391.2837 (C_24_H_39_O_4_^−^)
	829.4921 [M + HCOO]^−^	829.4944	−2.770	C_43_H_73_O_15_^−^	7.5	
DP-3 and DP-4	797.5040 [M-H]^−^	797.5046	−0.713	C_43_H_73_O_13_^−^	7.5	651.4446 (C_37_H_63_O_9_^−^), 633.4341 (C_37_H_61_O_8_^−^), 457.3665 (C_30_H_49_O_3_^−^)
	843.5076 [M + HCOO]^−^	843.5100	−2.902	C_44_H_75_O_15_^−^	7.5	
DP-5 and DP-6	765.4802 [M-H]^−^	765.4784	2.412	C_42_H_69_O_12_^−^	8.5	619.4189 (C_36_H_59_O_8_^−^), 601.4085 (C_36_H_57_O_7_^−^), 457.3669 (C_30_H_49_O_3_^-^)
	811.4828 [M + HCOO]^−^	811.4838	−1.273	C_43_H_71_O_14_^−^	8.5	
DP-7 and DP-8	801.5026 [M-H]^−^	801.4995	3.889	C_42_H_73_O_14_^−^	6.5	655.4414 (C_36_H_63_O_10_^−^), 637.4308 (C_36_H_61_O_9_^−^), 493.3900 (C_30_H_53_O_5_^−^)
	847.5050 [M + HCOO]^−^	847.5050	0.044	C_43_H_75_O_16_^−^	6.5	
DP-9 and DP-10	979.5447 [M-H]^−^	979.5472	−2.574	C_48_H_83_O_20_^−^	7.5	817.4913 (C_42_H_73_O_15_^−^), 799.4808 (C_42_H_71_O_14_^−^), 671.4352 (C_36_H_63_O_11_^−^), 653.4234 (C_36_H_61_O_10_^−^), 491.3716 (C_30_H_51_O_5_^−^)
	1025.5494 [M + HCOO]^−^	1025.5527	−3.218	C_49_H_85_O_22_^−^	7.5	
DP-11 and DP-12	995.5386 [M-H]^−^	995.5421	−3.552	C_48_H_83_O_21_^−^	7.5	919.4866 (C_45_H_75_O_19_^−^), 757.4344 (C_39_H_65_O_14_^−^), 739.4238 (C_39_H_63_O_13_^−^),
	1041.5441 [M + HCOO]^−^	1041.5476	−3.375	C_49_H_85_O_23_^−^	7.5	611.3768 (C_33_H_55_O_10_^−^), 593.3664 (C_33_H_53_O_9_^−^), 575.3556 (C_33_H_51_O_8_^−^),
						431.3148 (C_27_H_43_O_4_^−^)
DP-13	961.5375 [M-H]^−^	961.5367	0.877	C_48_H_81_O_19_^−^	8.5	815.4757 (C_42_H_71_O_15_^−^), 799.4809 (C_42_H_71_O_14_^−^), 781.4704 (C_42_H_69_O_13_^−^),
	1007.5393 [M + HCOO]^−^	1007.5421	−2.814	C_49_H_83_O_21_^−^	8.5	653.4238 (C_36_H_61_O_10_^−^), 635.4134 (C_36_H_59_O_9_^−^), 491.3716 (C_30_H_51_O_5_^−^)

^a^ RDBs: ring plus double bonds.

**Table 2:** Summary of degradation behaviors of Re.

Degradation Studies	Exposure Conditions	DPs
Acidic hydrolysis	0.1 M HCl: MeOH (1:1, *v*/*v*) at 37 °C for 1, 3, and 6 h	DP-1, DP-2, DP-3, DP-4, DP-5, DP-6
Acidic hydrolysis	0.1 M HCl at 37 °C for 1, 3, and 6 h	DP-1, DP-2, DP-5, DP-6, DP-7, DP-8
Basic hydrolysis	1.0 M NaOH at 37 °C for 1, 3, and 6 h	DP-1, DP-2, DP-5, DP-6
Neutral hydrolysis	MeOH at 70 °C for 1, 7, and 10 d	-
Oxidation	30% H_2_O_2_ at RT for 1, 3, 6, and 8 h	DP-9, DP-10, DP-11, DP-12, DP-13
Thermal	50 °C for 5, 10, and 50 d	-
Humidity	90% humidity at RT for 10 d	-
Visible light	4500 lX h at RT with 15% humidity for 14, 30, and 55 d	-
UV light	200 W·h/m^2^ at RT with 15% humidity for 30 d	-

Note: RT, room temperature; h, hours; d, days; -, no degradation.

## Data Availability

The authors confirm that the data supporting the findings of this study are available within the article and its Supplementary Materials.

## Supplementary Materials

The following supporting information can be downloaded at: https://www.mdpi.com/article/10.3390/ijms252413231/s1.
