# Supplementary material for: Structural Elucidation and In Silico-Aided Toxicity Prediction of Forced Degradation Products of Ginsenoside Re Using Ultra-High-Performance Liquid Chromatography Equipped with a Diode Array Detector and Charged Aerosol Detector (UHPLC-DAD-CAD) and Liquid Chromatography Coupled to a High-Resolution Mass Detector (LC-HRMS)
Source: Int J Mol Sci. 2024 Dec 10;25(24):13231. doi: 10.3390/ijms252413231 (PMC11676588; doi:10.3390/ijms252413231)
Supplement: Supplementary file 1 [file ijms-25-13231-s001.zip › ijms-3355977-supplementary.pdf]

International Journal of *Molecular Sciences*

MDPI

Supplementary Materials:

Structural Elucidation and In Silico-Aided Toxicity Prediction of Forced Degradation Products of Ginsenoside Re Using Ultra-High-Performance Liquid Chromatography Equipped with a Diode Array Detector and Charged Aerosol Detector (UHPLC-DAD-CAD) and Liquid Chromatography Coupled to a High-Resolution Mass Detector (LC-HRMS)

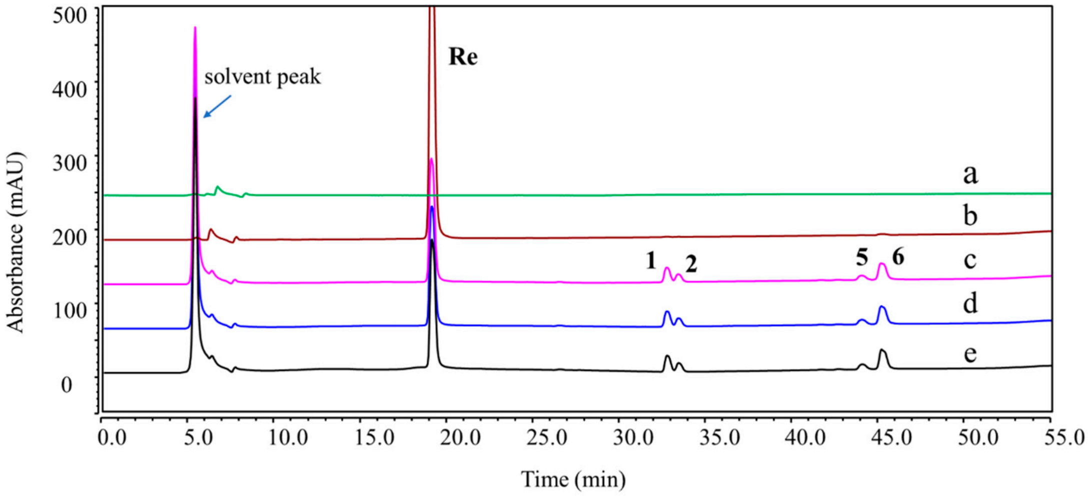

Figure S1. Overlay chromatograms of Re and its DPs under basic hydrolysis using UHPLC-UV (203 nm). **(a)** blank; **(b)** Re; **(c-e)** basic hydrolysis (1.0 M NaOH, 37℃) for 1, 3 and 6 h.

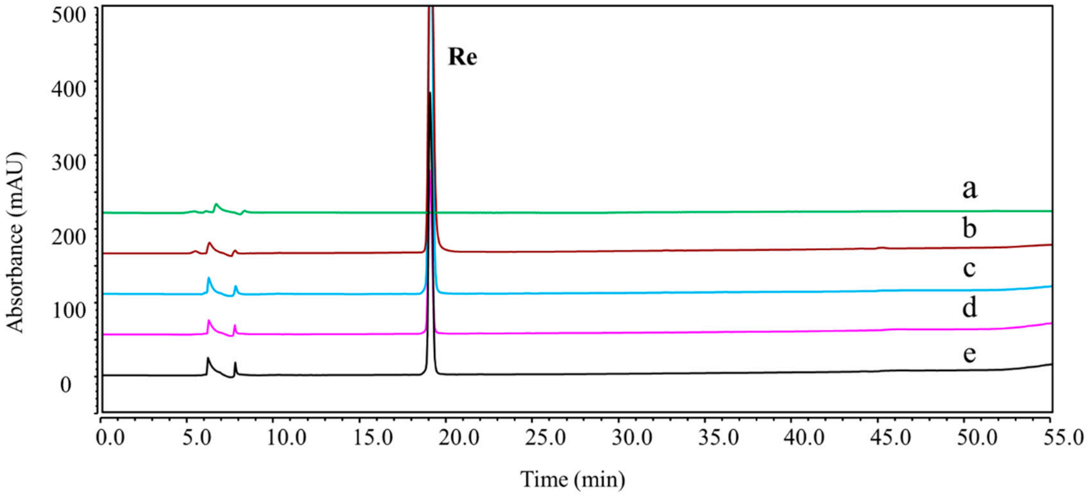

Figure S2. Overlay chromatograms of Re and its DPs under neutral hydrolysis using UHPLC-UV (203 nm). **(a)** blank; **(b)** Re; **(c-e)** neutral hydrolysis at 70℃ for 1, 7 and 10 d.

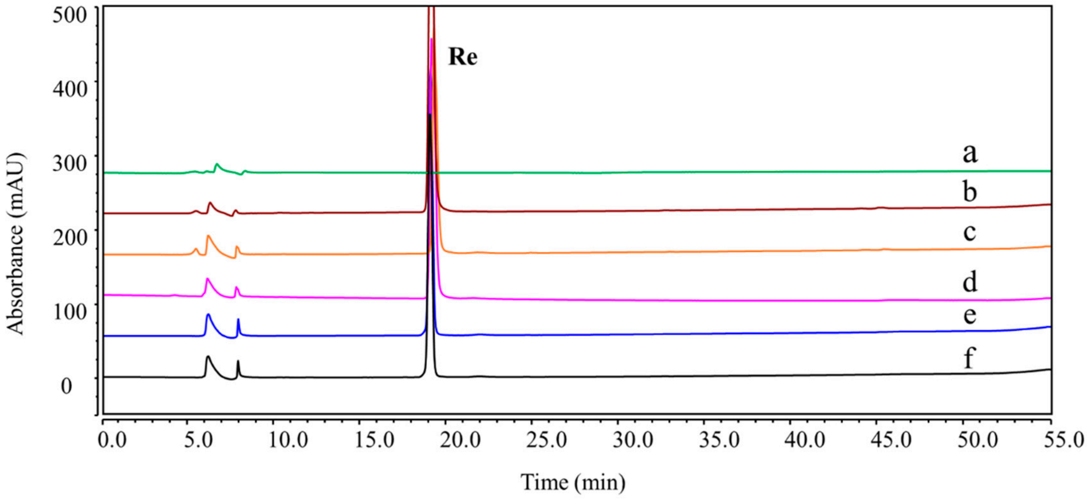

Figure S3. Overlay chromatograms of Re and its DPs exposed to high humidity and high temperature conditions (UHPLC-UV at 203 nm). **(a)** blank; **(b)** Re; **(c)** high humidity for 10 days (90% RH, 25℃, 10 d); **(d-f)** high temperature at 50℃ for 5, 10 and 50 d.

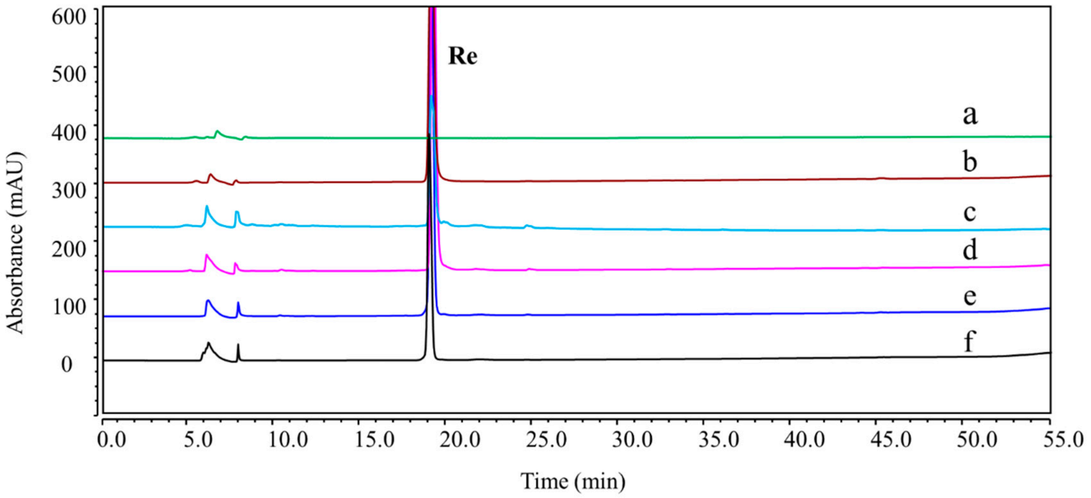

Figure S4. Overlay chromatograms of Re and its DPs exposed to photolysis condition (UHPLC-UV at 203 nm). **(a)** blank; **(b)** Re; **(c)** UV light for 30 d; **(d-f)** visible light for 14, 30 and 55 d.

A

B

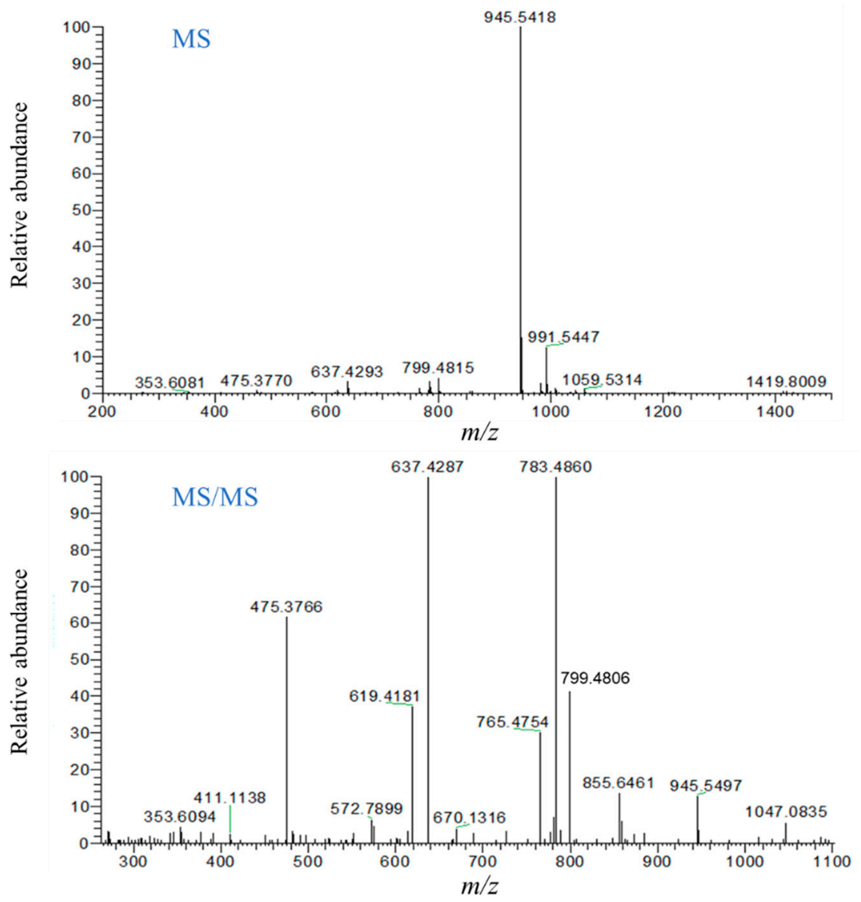

Figure S5. Mass spectrum of Re in ESI negative mode.

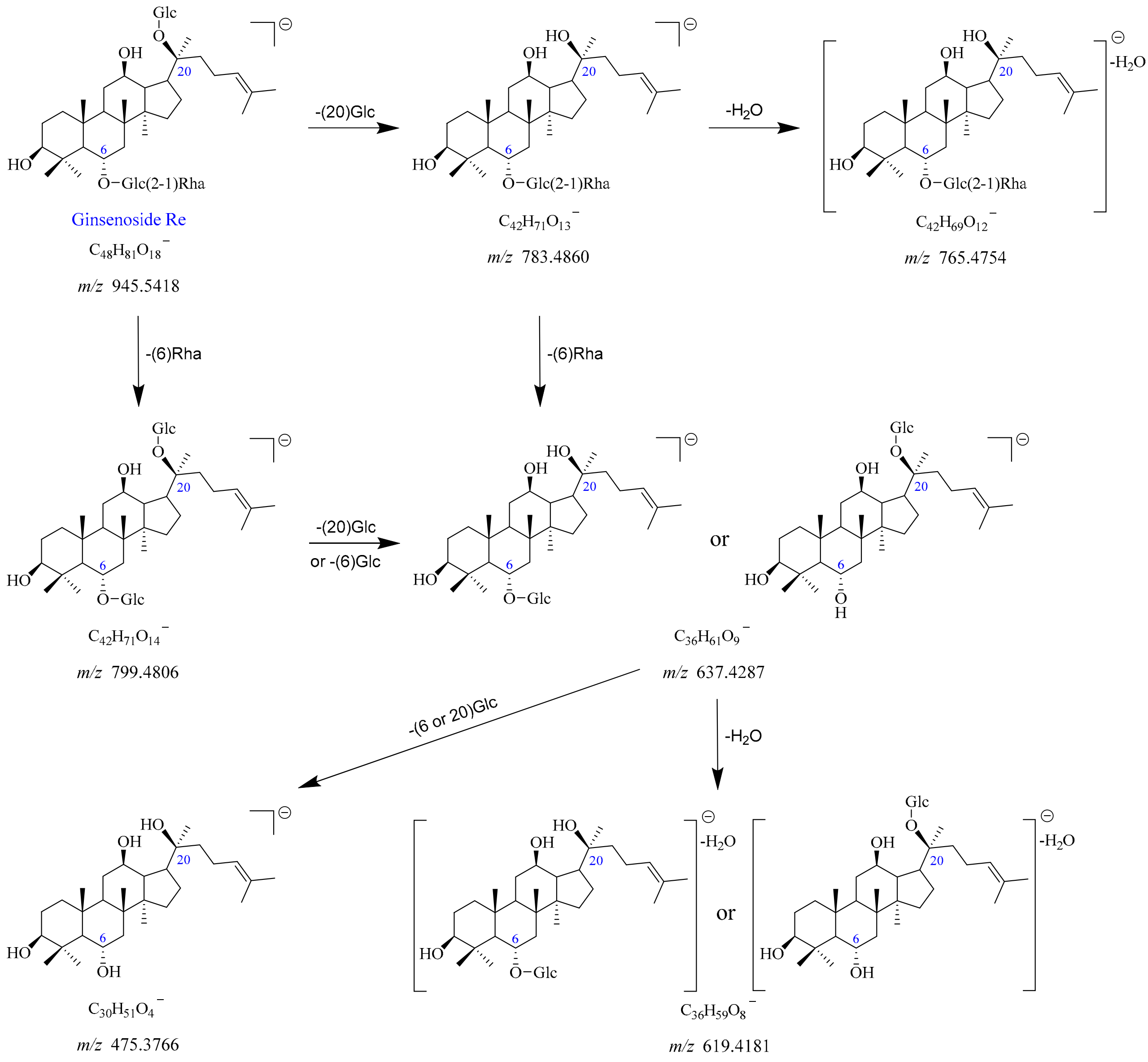

Figure S6. Proposed mass fragmentation pathway of Re.

A

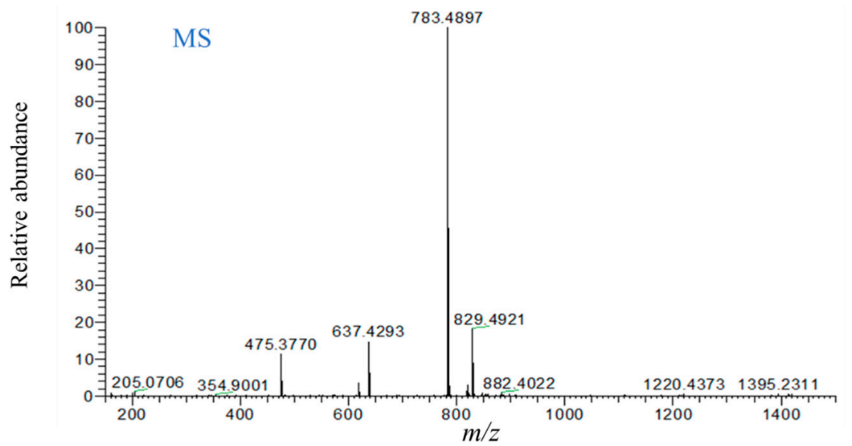

B

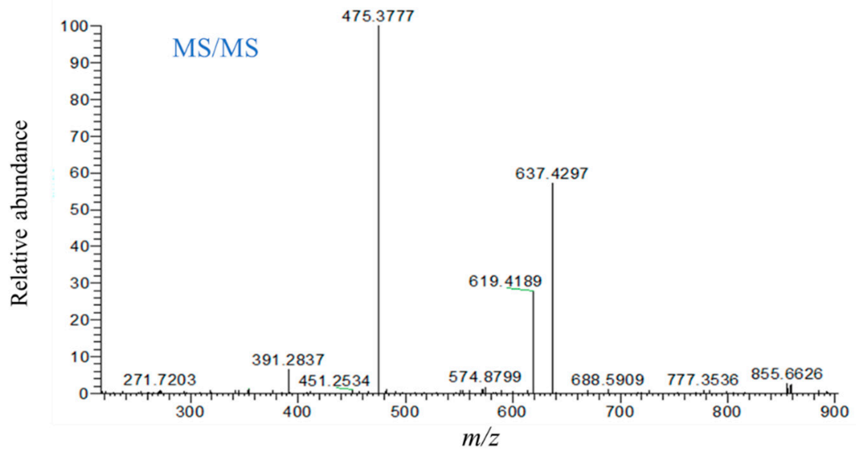

Figure S7. Mass spectrum of DP-1 & DP-2 in ESI negative mode.

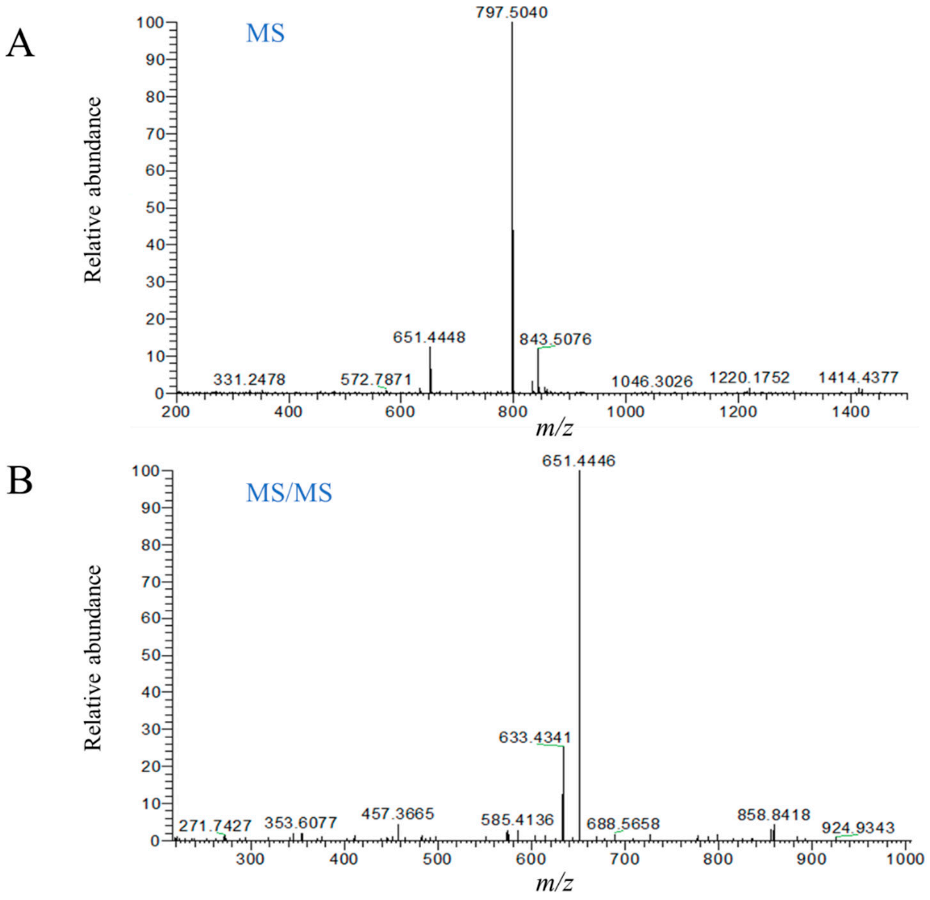

Figure S8. Mass spectrum of DP-3 & DP-4 in ESI negative mode.

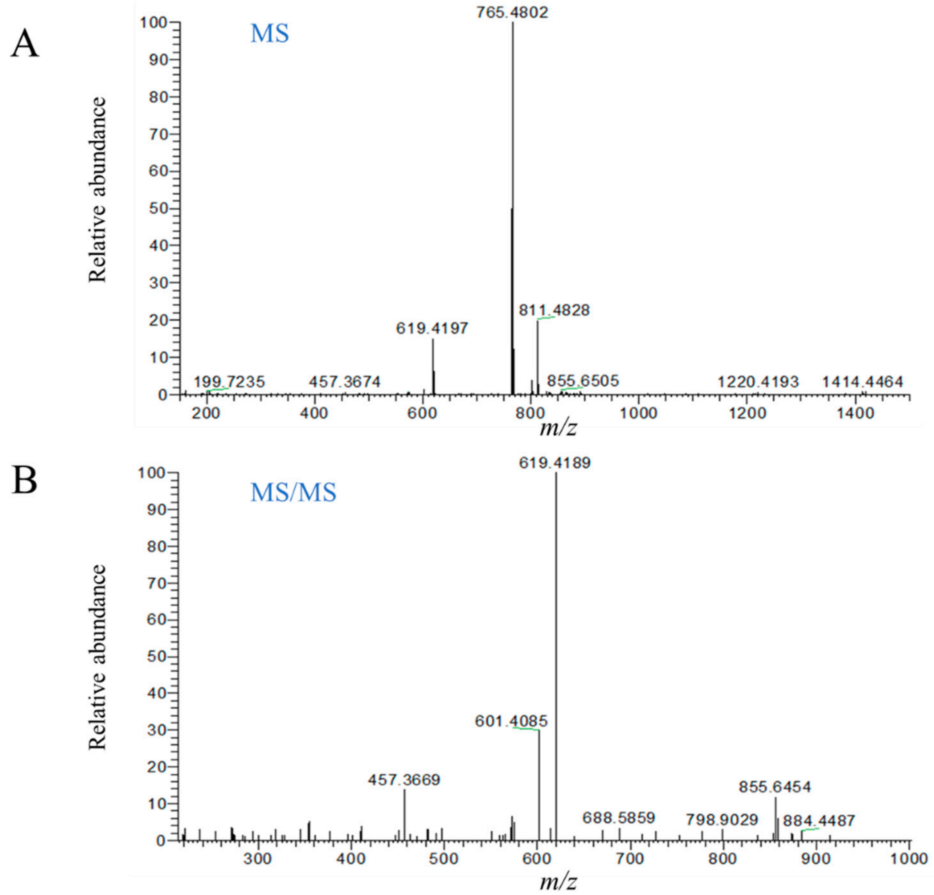

Figure S9. Mass spectrum of DP-5 & DP-6 in ESI negative mode.

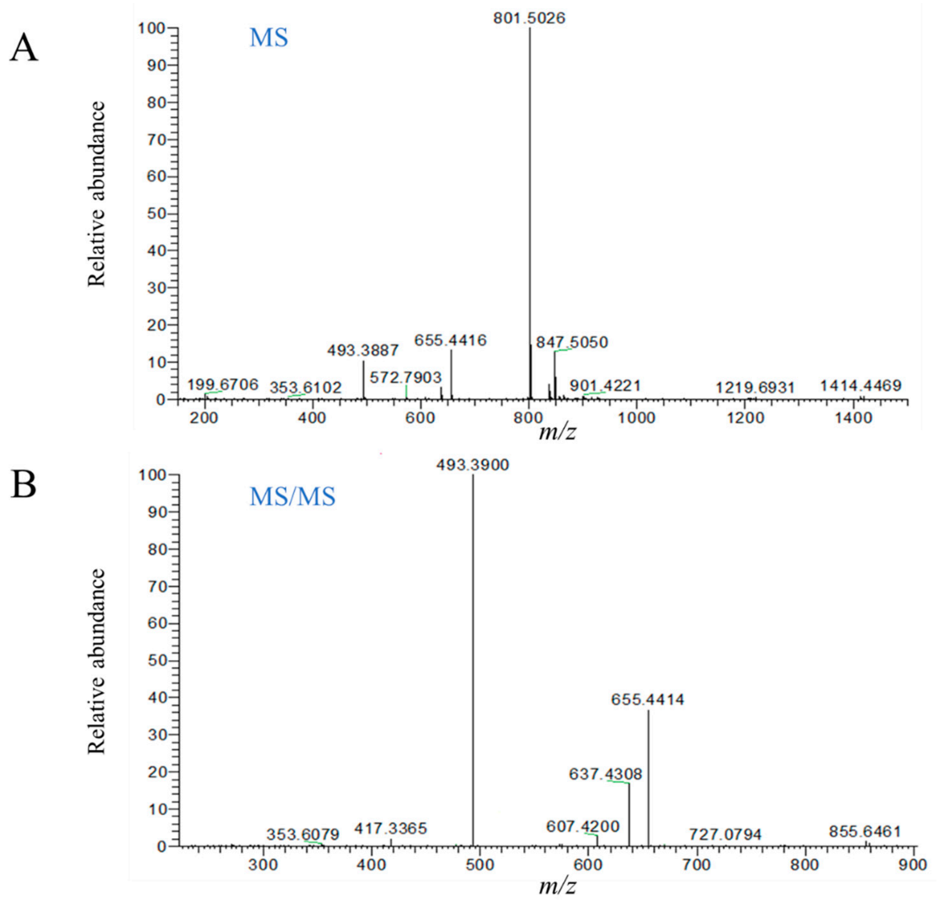

Figure S10. Mass spectrum of DP-7 & DP-8 in ESI negative mode.

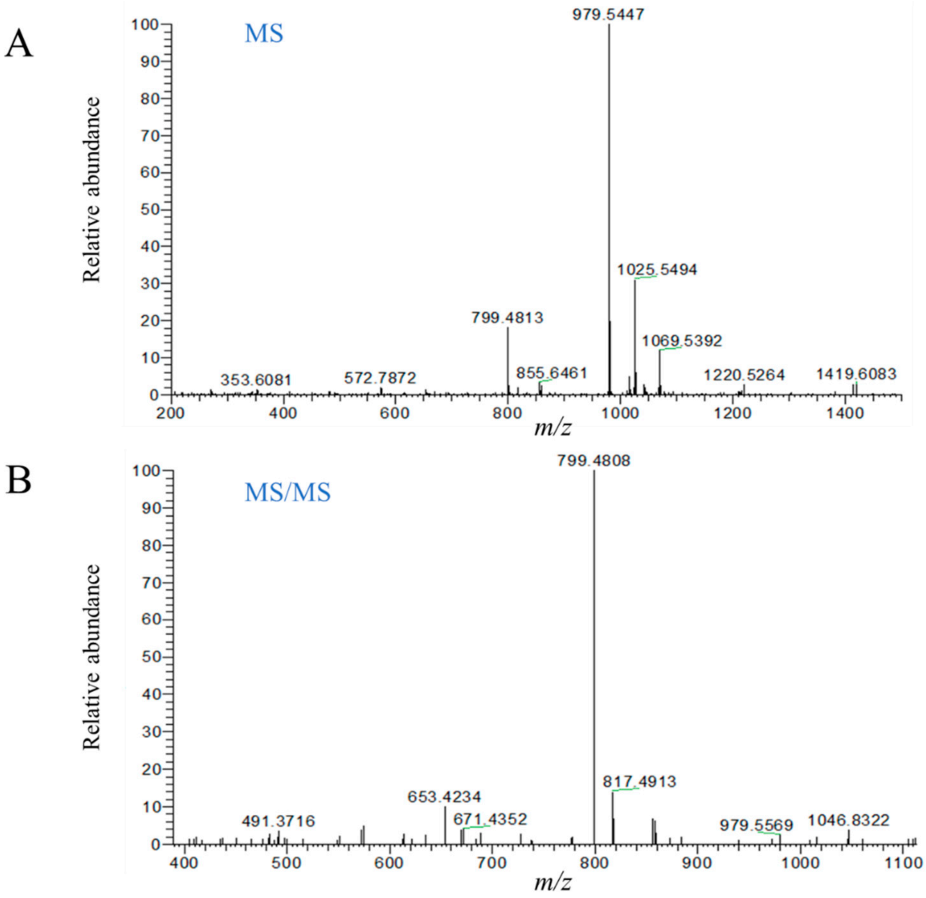

Figure S11. Mass spectrum of DP-9 & DP-10 in ESI negative mode.

A

B

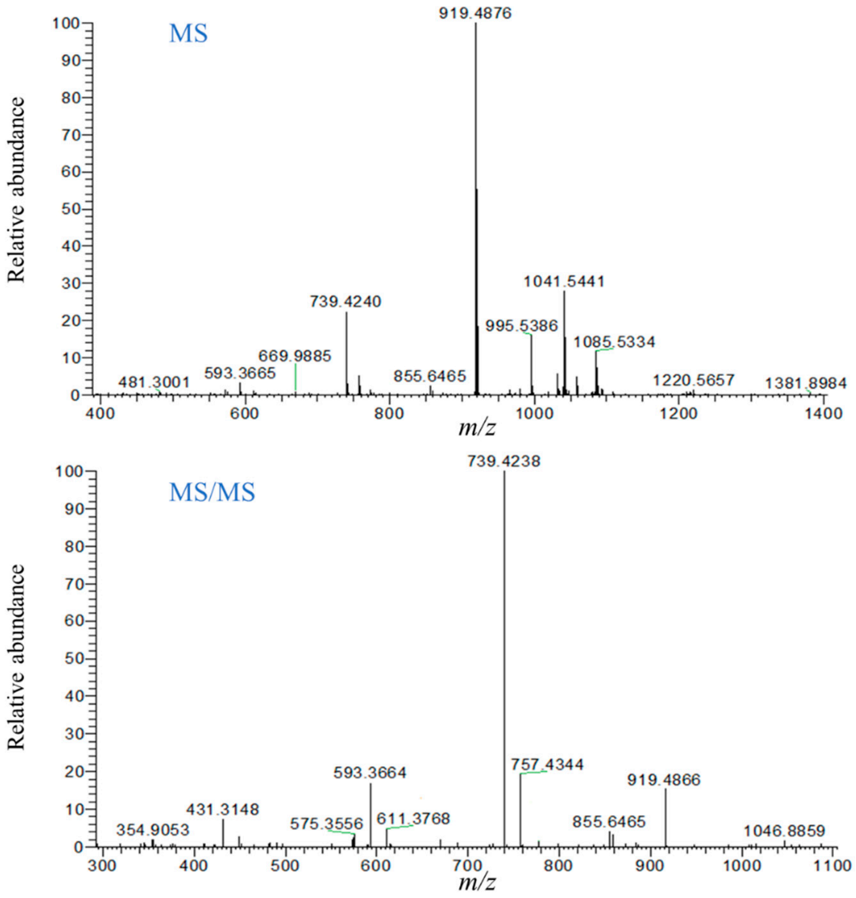

Figure S12. Mass spectrum of DP-11 & DP-12 in ESI negative mode.

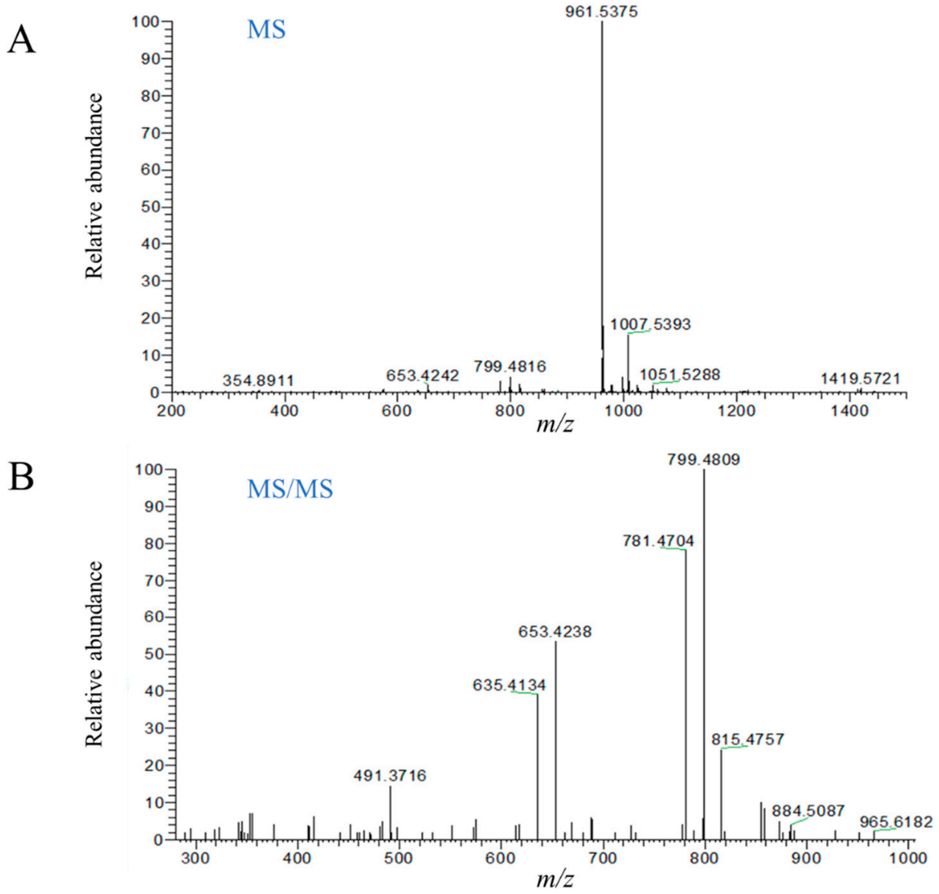

Figure S13. Mass spectrum of DP-13 in ESI negative mode.

Table S1. *In silico* toxicity predictions of Re and its DPs by Derek Nexus software.

**Alert structures / Toxicity**	**Re and its degradation products**															
	**Re**		**DP-1 and DP-2**		**DP-3 and DP-4**		**DP-5 and DP-6**		DP-7 and DP-8		DP-9 and DP-10		DP-11 and DP-12		DP-13	
**Carcinogenicity**	-	-	-	-	-	-	-	-	-	-	-	-	Carcinogenicity	Oxidising agent	-	-
Genotoxicity	-	-	-	-	-	-	-	-	-	-	-	-	Mutagenicity in vitro	Hydroperoxide	-	-
Irritation	Skin irritation/ corrosion	Terpenoid	Skin irritation/ corrosion	Terpenoid	Skin irritation/ corrosion	Terpenoid	Skin irritation/ corrosion	Terpenoid	-	-	-	-	Skin irritation/ corrosion	Hydroperoxide or peracid	-	-
													Irritation (of the eye)	Alkyl hydroperoxide		
													Irritation (of the respiratory tract)	Alkyl hydroperoxide		
Miscellaneous endpoints	-	-	-	-	-	-	-	-	-	-	-	-	-	-	-	-

Neurotoxicity	-	-	-	-	-	-	-	-	-	-	-	-	-	-	-	-
Organ toxicity	-	-	-	-	-	-	-	-	-	-	-	-	Hepato-toxicity	Organic peroxide	-	-
Reproductive toxicity	-	-	-	-	-	-	-	-	-	-	-	-	-	-	-	-
Respiratory sensitisation	-	-	-	-	-	-	-	-	-	-	-	-	-	-	-	-
Skin sensitisa-tion	-	-	-	-	-	-	-	-	-	-	-	-	Skin sensi-tization HPC	HPC08_1: Class 8: Organic peroxides	-	-

Table S2. *In silico* prediction of drug metabolism properties for Re and its DPs by Meteor Nexus software.

Re and its DPs	Biotransformation Name	Phase	Enzyme
Re	Allylic hydroxylation	I	CYP450 [a]
DP-1 and DP-2	Allylic hydroxylation	I	CYP450
	Epoxidation of 1,1,2-trisubstituted alkenes	I	CYP450
DP-3 and DP-4	Allylic hydroxylation	I	CYP450
	Epoxidation of 1,1,2-trisubstituted alkenes	I	CYP450
	Oxidative *O*-demethylation	I	CYP450
DP-5 and DP-6	Allylic hydroxylation	I	CYP450
	Epoxidation of 1,1,2-trisubstituted alkenes	I	CYP450
	Hydroxylation of methyl carbon adjacent to an aliphatic ring	I	CYP450
DP-7 and DP-8	Hydroxylation of terminal methyl	I	CYP450
	Hydroxylation of methyl carbon adjacent to an aliphatic ring	I	CYP450
	Glucuronidation of primary and secondary aliphatic and benzylic alcohols	II	UGT [b]
DP-9 and DP-10	Hydroxylation of methyl carbon adjacent to an aliphatic ring	I	CYP450
	Glucuronidation of primary and secondary aliphatic and benzylic alcohols	II	UGT
DP-11 and DP-12	Hydroxylation of methyl carbon adjacent to an aliphatic ring	I	CYP450
	Glucuronidation of primary and secondary aliphatic and benzylic alcohols	II	UGT
DP-13	Vicinal diols from epoxides	I	EH [c]

Note: [a] CYP 450, cytochrome P450 enzyme system; [b] UGT, uridine diphosphate glucuronate transferase; [c] EH, epoxide hydrolases.
